# Viral replication and interferon responses in bronchial epithelia is enhanced by Th17 cells

**DOI:** 10.1093/ajrcmb/aanag070

**Published:** 2026-03-27

**Authors:** Weston T Powell, Tomasz J Janczyk, Patricia C Dela Cruz, Basilin Benson, Nyssa B Samanas, Elizabeth R Vanderwall, Camille R Gates, Lucille M Rich, Teal S Hallstrand, Matthew C Altman, Jason S Debley

**Affiliations:** Center for Respiratory Biology and Therapeutics, Seattle Children’s Research Institute, Seattle, WA, United States; Department of Pediatrics, Division of Pulmonary and Sleep Medicine, Seattle Children’s Hospital, University of Washington, Seattle, WA, United States; Systems Immunology Division, Benaroya Research Institute at Virginia Mason, Seattle, WA, United States; Center for Respiratory Biology and Therapeutics, Seattle Children’s Research Institute, Seattle, WA, United States; Division of Allergy and Infectious Diseases, University of Washington School of Medicine, Seattle, WA, United States; Systems Immunology Division, Benaroya Research Institute at Virginia Mason, Seattle, WA, United States; Center for Respiratory Biology and Therapeutics, Seattle Children’s Research Institute, Seattle, WA, United States; Center for Respiratory Biology and Therapeutics, Seattle Children’s Research Institute, Seattle, WA, United States; Center for Respiratory Biology and Therapeutics, Seattle Children’s Research Institute, Seattle, WA, United States; Center for Respiratory Biology and Therapeutics, Seattle Children’s Research Institute, Seattle, WA, United States; Center for Lung Biology, Division of Pulmonary, Critical Care and Sleep Medicine, University of Washington, Seattle, WA, United States; Systems Immunology Division, Benaroya Research Institute at Virginia Mason, Seattle, WA, United States; Division of Allergy and Infectious Diseases, University of Washington School of Medicine, Seattle, WA, United States; Center for Respiratory Biology and Therapeutics, Seattle Children’s Research Institute, Seattle, WA, United States; Department of Pediatrics, Division of Pulmonary and Sleep Medicine, Seattle Children’s Hospital, University of Washington, Seattle, WA, United States

To the Editor:

Infections with human rhinoviruses are the most common trigger of asthma exacerbations in children and adults.^1^ Asthma endotypes range from type 2 (T2) to non-T2.^2^ Although allergic airway inflammation in T2 asthma is well studied, the mechanisms of airway inflammation and signaling in non-T2 endotypes are poorly understood, especially during viral infections. Non-T2 asthma is hypothesized to be in part driven by an interleukin (IL)-17–mediated pathway through Th17 cells causing neutrophilic inflammation.^2^ Th17 cells arise in response to bacterial and fungal antigens, function in autoimmune and inflammatory conditions, and coordinate neutrophilic inflammation during human rhinovirus infections.^3^ Coculture of Th17 cells with bronchial fibroblasts leads to an increase in IL-17 production, as well as IL-6, IL-1β, and IL-23; however, effects on epithelial gene expression were not investigated.^4^ Increased Th17 cells and related cytokines have been observed in the bronchial mucosa of adults with severe asthma with frequent exacerbations in association with increased epithelial neutrophils.^5^ Accordingly, airway epithelial-Th17 cell crosstalk may underpin a pathogenic mechanism in non-T2 asthma, particularly in the setting of viral-triggered exacerbations. However, how Th17 cell crosstalk with the airway epithelium alters innate immune and inflammatory responses to rhinovirus infection remains uninvestigated. We hypothesized that Th17 cells in coculture with human bronchial epithelial cells (BECs) from children with asthma would alter innate immune responses by both Th17 cells and BECs following rhinovirus infection.

Primary BECs from 10 children with asthma undergoing elective surgery (Table 1) were obtained under Seattle Children’s Hospital Institutional Review Board approval as previously described.^6^ Primary healthy donor Th17 cells (STEMCELL; 25 000 cells/mL) were cocultured basolaterally with differentiated, organotypic, pseudostratified BECs for 5 days. Th17 cell identity was confirmed by flow cytometry as CD4+ CXCR3-CCR6+ with positive intracellular staining for RORγT and IL-17A. BECs were infected apically with human rhinovirus A16 (ATCC) at multiplicity of infection 0.5 on day 4 of coculture.^6^ Th17 cells were removed for RNA isolation 24 hours after infection. To model the effector phase of Th17-mediated asthma, healthy primary neutrophils (iQ Biosciences; 25 000 cells/mL) were added basolaterally to BECs to emulate neutrophilic infiltration characteristic of non-T2 airway inflammation. BEC RNA and media were collected 24 hours after neutrophil addition. Protein concentrations of secreted IL-1β and IFN-α were measured via Luminex Assay (R&D). RNA was sequenced with paired-end 53-base reads with quality control and gene counts generated, as previously described,^6^ and available at NCBI GEO GSE314289. Viral quantification in reads unmapped to the human reference genome (GRCh38) was performed using the Salmon (v1.10.2) quasi-mapping algorithm against a custom viral reference of 42 complete viral genomes.^7^ Differentially expressed genes and protein levels were identified using mixed effect linear models via *kimma* (model syntax: gene_expression ∼ condition*infection and a random effect for donor). We identified 4030 genes in BECs and 1899 genes in Th17 cells at a false discovery rate < 0.05. Weighted gene co-expression network analysis identified 10 modules within BECs and 9 modules in Th17 cells. We used the GO_BP, KEGG, Reactome, Biocarta, and MSigDB Hallmark genesets for enrichment analysis to guide module annotation. Detailed methods and results, including schematics for the experimental design and flow cytometry characterization, are available on DataDryad at http://datadryad.org/share/LINK_NOT_FOR_PUBLICATION/-_yt2INndql32Qa2-PkT2GRVqwfMjSoEMHuxG6Qvo14.

**Table 1:** Demographics of BEC donors for coculture experiments; mean (range) presented.

Sex (% Female)	30%
**Age (mean, range)**	11.2 (6.32-17.85)
**FeNO (mean, range)**	20.3 (6-54)
**FEV1 % (mean, range)**	102.1 (86-117)
**FEV1/FVC (mean, range)**	0.82 (0.75-0.92)
**IgE Level (mean, range)**	266.8 (3-1204)
**% with history of severe exacerbation (ER visit, hospitalization or steroid use)**	90%
**% with T2-high (FeNO > 35 ppb in steroid-naive or > 20 ppb on inhaled corticosteroid, or absolute eosinophils > 300)**	40%

A BEC “Interferon Signaling” module composed of primarily type II interferon signaling and viral response genes showed synergistic increased expression from Th17 priming and viral infection with mean expression unchanged in Th17-primed BECs preinfection (Figure 1A). Two modules in BECs, “MAP-Kinase Signaling and Stress Response” and “TNF-signaling,” demonstrated increased expression in an additive fashion because of both Th17 priming and RV16 infection (Figure 1A). Three modules in BECs demonstrated decreased expression because of both Th17 priming and viral infection (“Cilia Structure/Function and Cellular Metabolism,” “Macromolecule Metabolism,” and “Ribosome Subunits”) (Figure 1A).

**Figure 1:**
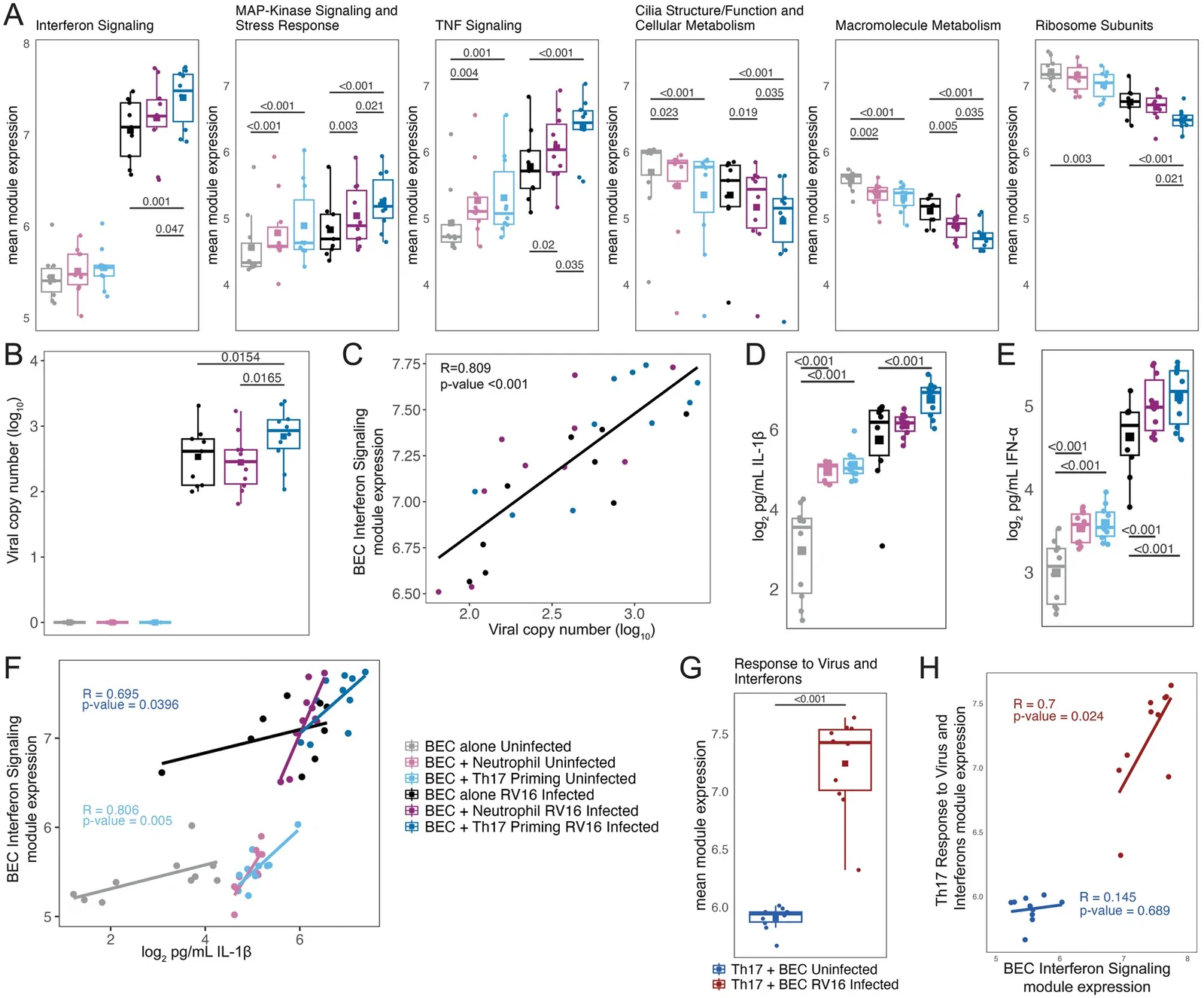
Th17 Priming alters gene expression in BECs and viral copy number, increases cytokine secretion and induces transcriptional responses in Th17 cells. **A**. Five modules identified in BECs with additive differential gene expression from Th17 coculture and RV-16 infection and 1 module with mean gene expression unchanged due to coculture under uninfected conditions, but synergistic increase in expression following infection of BECs primed with Th17 coculture. **B**. Viral copy number in uninfected and infected BECs is higher with Th17 priming. **C**. Correlation of viral copy number with expression of the interferon signaling module in BECs across all infected culture conditions. **D**. Secreted protein measurement of cytokines from each culture and infection condition were measured by Luminex protein quantification for IL-1β and **E**. IFN-α. **F**. Correlation of IL-1β protein levels in supernatant with BEC interferon signaling module. **G**. Mean gene expression of response to virus and interferons module in Th17 cells cocultured with uninfected BECs for 5 days or for 5 days with BECs infected on day 4 of coculture. **H**. Correlation of interferon signaling module in BECs with interferon and viral response module in Th17 cells with (red) and without RV-16 infection (blue). All modules and protein levels showed statistically significant differential expression when comparing uninfected to RV-16–infected conditions. Multiple hypothesis testing correction was performed using the Benjamini-Hochberg procedure. Pearson correlation of module expression in BECs to module expression in Th17 cells, for BEC module expression to viral copy number, and for protein level to module expression was performed. For figure legibility, only the statistical comparisons within uninfected or within RV-16 infected conditions are shown.

Viral load was increased in BECs primed with Th17 cells as compared to primary BECs alone or BECs cocultured with neutrophils (Figure 1B). Viral genomes were only detected in RNA isolated from BECs, and not in RNA isolated from neutrophils or Th17 cells. The expression of the BEC Interferon Signaling module correlated with viral genome copy number (Figure 1C). Measurement of secreted cytokines showed increased secretion of IL-1β from Th17-primed BECs following RV-16 infection (Figure 1D). IFN-α levels were increased by coculture with Th17 cells and neutrophils, as well as RV-16 infection (Figure 1E). Protein levels of IL-1β correlated with expression of the BEC Interferon Signaling module (Figure 1F).

The module with the greatest increase in expression in Th17 cells following infection of BECs was “Response to Virus and Interferons” (Figure 1G). Expression of the BEC module for Interferon Signaling and the Th17 module for Response to Virus and Interferons did not correlate in the uninfected state, but following RV infection expression increased in both cell populations and expression was highly correlated across each coculture pair (Figure 1H).

We observe that Th17 coculture alters gene expression in primary BECs from children with asthma and modifies the epithelial response to viral infection as demonstrated by synergistically increased expression of interferon signaling in BECs with greater epithelial viral load. Th17 cells upregulated interferon-response genes when cocultured with RV-infected epithelium. Enhanced interferon signaling, together with elevated IL-1β secretion, indicate an amplified innate epithelial response to RV-16 after Th17 priming.

Our study reveals that Th17 cells shape epithelial and immune responses to rhinovirus infection in the pediatric asthmatic airway by enhancing viral replication and amplifying interferon and proinflammatory gene programs in both epithelium and Th17 cells. In addition, our demonstration that Th17 coculture enhanced expression of tumor necrosis factor and MAP-kinase signaling modules underscores the inflammatory amplification role of Th17–epithelial interactions leading to epithelial injury and remodeling, which are key features of asthma pathogenesis.^8-10^ In parallel, Th17 coculture decreased expression of cellular metabolism and macromolecular metabolism genes post RV16 infection. Our finding of increased viral copy number with Th17 coculture may reflect a susceptibility to RV16 infection as we observed decreased expression of antiviral genes such as *RTP4* and *LTF* and immune effector genes *TLR4* and *IRAG2* in BECs with Th17 coculture. Our study has several limitations including a modest sample size and the use of nonautologous adult peripheral Th17 cells that may not fully reflect mucosal resident Th17 cells. We also did not directly stimulate Th17 cells or neutrophils before coculture and future studies are necessary to investigate how Th17 or neutrophil activation alters BEC responses. The sample size prevents correlation with donor clinical measures such as asthma severity or T2-high/non-T2 phenotype, and autologous Th17 cells from asthmatic children could show greater dysregulation. Furthermore, our study did not include BECs from donors without asthma and future studies are needed to investigate how BECs from donors with and without asthma may lead to different immune responses from cocultured immune cells. Despite these limitations, the use of primary, organotypic epithelial cultures from well-characterized children with asthma represents a major strength, providing physiologically relevant insights that extend beyond transformed cell lines. By identifying epithelial–Th17 interactions as critical regulators of viral responses, our findings advance mechanistic understanding of non-T2 asthma and highlight novel pathways that may be targeted to prevent or attenuate severe viral-induced exacerbations in children, particularly in children who have not responded to therapies targeting T2 inflammation.

## Supplementary Material

aanag070_Supplementary_Data
